# In Vitro Effect of Flavonoids on Basophils Degranulation and Intestinal Epithelial Barrier Damage Induced by ω-5 Gliadin-Derived Peptide

**DOI:** 10.3390/foods11233857

**Published:** 2022-11-29

**Authors:** Shuangshuang Wu, Ranran Zhang, Yaran Liu, Jinyan Gao, Yong Wu, Changchun Tu, Hongbing Chen, Juanli Yuan

**Affiliations:** 1 School of Pharmacy, Nanchang University, Nanchang 330006, China; 2 Fuzhou Medical College, Nanchang University, Fuzhou 344000, China; 3 Jining First People’s Hospital, Jining 272002, China; 4 School of Food Science and Technology, Nanchang University; Nanchang 330047, China; 5 Sino-German Joint Research Institute, Nanchang University, Nanchang 330047, China; 6 State Key Laboratory of Food Science and Technology, Nanchang University, Nanchang 330047, China

**Keywords:** flavonoids, wheat food allergy, ω-5 gliadin, degranulation, tight junction

## Abstract

Flavonoids have antioxidant, anti-inflammatory and immunomodulatory properties, and may alleviate food allergic reactions and intestinal inflammation induced by ω-5 gliadin, a main allergen of wheat food allergy in children. In this study, a human basophil KU812 cell degranulation model and a Caco-2 monolayer cell model were constructed in vitro to evaluate the effects of four flavonoids on the allergenicity of ω-5 gliadin peptides and ω-5 gliadin peptide-induced barrier damage in Caco-2 intestinal epithelial monolayers. The results show that baicalein, luteolin, isorhamnetin and naringenin can significantly inhibit the degranulation of KU812 cells stimulated by ω-5 gliadin-derived peptide P4 and the release of IL-6 and TNF-α. In addition, the four flavonoids significantly inhibited the ω-5 gliadin-derived peptide P4 to induce the release of IL-6, IL-8 in Caco-2 cells, inhibited the release of zonulin, and significantly increase the expression of tight junction proteins Occludin and ZO-1 in the Caco-2 cell monolayer. In conclusion, baicalein, luteolin, isorhamnetin and naringenin inhibit degranulation stimulated by wheat allergen and enhance intestinal barrier functions, which supports the potential pharmaceutical application of the four flavonoids treatment for wheat food allergy.

## 1. Introduction

Wheat (Triticum aestivum) is one of the commonly grown crops worldwide. Due to its versatility, wheat can be processed into various foods and drinks. However, wheat also contains many allergenic proteins, divided into four classes: albumins, globulins, gliadins and glutenins [1]. Gliadins and glutenins are known as gluten and constitute up to 85% of wheat proteins [1]. Depending on the underlying immunologic mechanisms, wheat proteins can lead to a wide range of disorders: T cell-mediated autoimmune disease (celiac disease), and IgE-mediated wheat allergy and nonceliac gluten sensitivity [2]. The clinical manifestations of IgE-mediated wheat allergy can be different depending on the route of allergen exposure [1]. Inhalation-induced wheat allergy can cause baker’s asthma and rhinitis, which usually occurs in adult patients with occupational exposure to flour [3]. Sensitization to wheat by ingestion can produce food allergy symptoms, such as nausea, abdominal pain, urticaria, angioedema and bronchial obstruction, or severe systemic anaphylaxis [3], which is more common in children [4], and most children with wheat allergy suffer from moderate to severe atopic dermatitis. Wheat-dependent, exercise-induced anaphylaxis (WDEIA) is also a manifestation of wheat allergy, which is a severe allergic reaction induced by wheat ingestion and subsequent physical exercise [3].

ω-5-gliadin has been identified as the main allergen of wheat food allergy in children [5,6], and has the highest specificity in the diagnosis of IgE-mediated wheat allergy, especially in children with a history of allergy [7]. ω-5 gliadin is also reported to be the major allergen of WDEIA, and its major IgE-binding epitope sequence QQX1PX2QQ (X1 is I, L, F, S or Y; X2 is Q and E) has been obtained from patients with WDEIA from Japan and Europe [8,9,10], and these epitopes are also present in pediatric patients with wheat allergy with urticaria or anaphylactic shock [11].

The current treatment for wheat allergy is dietary avoidance [1,12]. However, because wheat is widely used in various food products, such as noodles, breads, cakes and seasoning soy sauce, the strict avoidance of wheat is difficult to reach in daily life. Inadvertent exposure to small traces may also cause serious allergic reactions; therefore, new treatments to relieve the effects of inadvertent exposure to wheat are needed [2]. Studies have shown traditional Chinese medicine (TCM) formulas and active compounds from TCM suppressed allergen-specific IgE and mast cell/basophil activation, and modulated cytokine profiles, has potential as effective complementary therapy for food allergy [13].

Flavonoids are a group of naturally-occurring polyphenolic compounds that are widely present in vegetables, fruits, and herbs, and are secondary metabolites of plants [14], with antioxidant [15,16], anti-inflammatory [17,18], immunomodulatory [19,20] and anti-cancer [21] properties. Based on anti-inflammatory and immunomodulatory activities, the effect of flavonoids on allergic diseases has been widely studied, such as allergic asthma, allergic rhinitis and atopic dermatitis [22]. In addition, several studies have reported the benefits of flavonoids in ovalbumin-induced [23] and peanut-induced food allergy [24], but little has been reported regarding the effect of flavonoids on wheat allergy. 

The intestinal epithelial barrier serves a major role in defense against intraluminal toxins, bacteria, and antigens. When intestinal barrier integrity is disrupted, sensitization to food allergens can occur, causing a T-helper (Th) 2-mediated immune response, which results in antigen-specific IgE antibodies binding to Fcε receptors on mast cells and basophils, and cross-link with the allergens, initiating the degranulation process, releasing β-hexosaminidase (β-Hex), histamine and other preformed mediators, which causes food allergy symptoms [25]. Therefore, the enhancement of intestinal barrier function and inhibition of degranulation in effector cells help to attenuate the food allergy. 

Based on previous studies, the flavones baicalein [23,26] and luteolin [27,28], the flavonol isorhamnetin [29] and the flavanone naringenin [30,31] are four flavonoids with anti-inflammatory and immunomodulatory activities. In the study, we selected the four flavonoids and investigated the effects of these flavonoids on degranulation in ω-5 gliadin peptide-stimulated KU812 cells, and ω-5 gliadin peptide-induced inflammatory response and barrier damage in Caco-2 intestinal epithelial monolayers. We further explored the possibility of these flavonoids as nursing interventions and complementary therapy for wheat food allergy.

## 2. Materials and Method

### 2.1. In Vitro Simulation of Gastrointestinal Digestion of Gliadin in Infants and Young Children

In vitro simulated digestion was performed according to Minekus M [32], Brodkorb A [33] and Dupont D [34] with some modifications. According to Minekus M [32], simulated gastric fluid (SGF) and simulated intestinal fluid (SIF) were prepared. Wheat gliadin, pepsin from porcine stomach mucosa and pancreatin were purchased from Sigma-Aldrich (St. Louis, MO, USA). 10 mL of wheat gliadin solution (40 mg gliadin mixed with 10 mL distilled water) was mixed with 7.5 mL of SGF solution, 1.6 mL pepsin solution of 3125 U/mL, 5 mL of 0.3 M CaCl_2_ and 0.695 mL of water. Subsequently, the pH was adjusted to 3.0 with 1 M HCl, and incubated at 37 °C for 2 h on a shaker to conduct simulated gastric digestion in vitro. After the incubation, the gastric digestion was ended by adjusting the pH to 7.0 with 1 M NaHCO_3_. Finally, gastric digestion products were detected by tricine-sodium dodecyl sulphate-polyacrylamide gel electrophoresis (Tricine-SDS-PAGE) and reverse phase-high performance liquid chromatography (RP-HPLC).

Add 12.5 mL of SIF solution, 5 mL of pancreatin solution (80 U/mL), 40 μL of 0.3 M CaCl_2_, 1 mL of bile salt solution (50 mM), and 1.3 mL of distilled water to the tube after gastric digestion and mix thoroughly. Then, the pH of samples was adjusted to 7.0 with 1 M HCl and incubated at 37 °C for 2 h on a shaker for in vitro simulated intestinal digestion. After the incubation, the digestive tube was placed in a 100 °C water bath for 5 min to end the intestinal digestion. Intestinal digestion products were finally detected by Tricine-SDS-PAGE and RP-HPLC. 

### 2.2. Tricine-SDS-PAGE and RP-HPLC Analysis

To 10 μL of digestion products, 10 μL of Tricine–SDS–PAGE loading buffer was added and heated in boiling water bath (100 °C) for 5 min. Each sample (15 μL) was loaded onto Tricine SDS polyacrylamide gel (4% stacking gel; 10 % spacer gel; 16.5 % separating gel) and separated by electrophoresis for 1 h at 30 V and 2 h at 100 V. After electrophoresis, the gel was fixed with methyl alcohol/acetic acid (5/1, *v*/*v*) solution for 15 min. After fixing, gel was stained with 0.05% (*w*/*v*) Coomassie Blue G-250 in 50% (*v*/*v*) methyl alcohol for 15 min, and destained by 10% (*v*/*v*) acetic acid. The band intensities on gel were analyzed by G:Box F3 Gel Documentation System(Syngene, Cambridge, UK).

Aliquots of 30 µL samples were analyzed using RP-HPLC. A LC-20AT model system (Shimadzu, Kyoto, Japan) and a C18 column (4.6 mm i.d. × 250 mm, 5 mm, Inertsil WP300; GL Sciences, Kyoto, Japan) were used. The analysis method was performed as described by Jun Lu et al. [35]

### 2.3. Screening and Synthesis of ω-5 Gliadin-Derived Peptides

The final products of static simulated infant gastrointestinal tract digestion in vitro were collected and analyzed peptides of ω-5 gliadin generated by in vitro digestion of gliadin using liquid chromatography-mass spectrometry (LC-MS). Samples analysis were carried out on Acquity UPLC I-class/VION IMS QTOF (Waters, Milford, MA, USA) in positive and negative ion mode respectively. A Waters ACQUITY UPLC^®®^ BEH C18 1.7 μm 2.1 × 100 mm Column (Waters, Milford, MA, USA) was used. The eluents used were as follows: (A) 0.1% formic acid–water (*v*/*v*), and (B) 0.1% formic acid-acetonitrile (*v*/*v*). The gradient elution procedure was: 0–3 min, 5–20% B; 3–10 min, 20–100% B; 10–12 min, 100% B; 12–15 min, 100–95% B; 15–19 min, 95% B. The flow rate was 0.4 mL/min, and the column temperature was 45 °C. The samples were operated at 210 nm. The mass spectrometry parameters were as follows: Cone voltage: 40 V, capillary voltage (positive and negative ion): 2000 V, desolvation temperature: 450 °C, desolvation gas: 900 L/h. MS mode acquisition was performed over the m/z range 50–1000.

The main allergen epitopes of ω-5 gliadin were obtained by referring to the literature [9,10,36] (Table 1). Combined with the amino acid sequence of ω-5 gliadin (439 amino acids in total, accession number: BAE20328.1) obtained from the GenBank database and the amino acid sequence of the digested product, allergenic epitope-bearing peptides of ω-5 gliadin were screened out.

The selected ω-5 gliadin-derived peptide sequences were sent to Shanghai Sangon Bioengineering Co., Ltd. (Shanghai, China) for synthesis, and the purity of the synthetic peptides was required to be no less than 95%.

### 2.4. Establishment of Serum Pools in Children with Wheat Allergy

The serum used in this experiment were provided by Peking Union Medical College Hospital, Beijing, China. The serum of 13 children with wheat allergy was mixed in equal volume to prepare a serum pool. The relevant information is shown in Table S1. The study was conducted in accordance with the Declaration of Helsinki, and the protocol was approved by the Ethics Committee of the Second Affiliated Hospital of Nanchang University (No. 20160309-018).

### 2.5. Cell Culture and Viability Testing

Human basophilic KU812 cells and Caco-2 human intestinal cells were obtained from the Chinese Academy of Sciences Cell Bank (Shanghai, China) and the American type culture collection Cell Bank (ATCC, Manassas, VA, USA), respectively. KU812 cells were cultured in RP1640 medium (Cellmax, Beijing, China) supplemented with 15% heat-inactivated fetal bovine serum (FBS) (Cellmax, China), 1% penicillin G and streptomycin at 37 °C (5% CO_2_ /95% air). Caco-2 cells were multiplied in Dulbecco’s Modified Eagle’s Medium (DMEM) complete medium (Cellmax, China), supplemented with 10% heat-inactivated FBS 1% penicillin G and streptomycin, 1% L-Glutamine and 1% non-essential amino acids at 37 °C in a 5% CO_2_ incubator. The medium was refreshed every 2 days. The cells were routinely subcultured at 80% confluence. All experiments were performed using Caco-2 cells from passages 28 to 30.

Cell viability was assessed by a Cell Counting Kit-8 (CCK-8; Beyotime Biot echnology, Shanghai, China) according to manufacturer’s instructions. Briefly, Cells seeded at a density of 1 × 10^5^/well (KU812 cells) or 2 × 10^4^/well (Caco-2 cells) in 96-well plates. After culturing for 24 h, cells were treated with different concentrations of tested substances for 24 h. The final concentrations of ω-5 gliadin-derived peptide in the medium were 5, 50, 150, 300 and 500 μg/mL.The final concentration of baicalein, luteolin, isorhamnetin and naringenin (Solarbio, Beijing, China) ranged from 1 μM to 100 μM. The cells were incubated for 24 h, then 10 μL of CCK-8 reagent was added to the cells for 2 h. The value of optical density was measured at 450 nm with a microplate reader (Bio-Rad, Hercules, CA, USA). Blanks (fresh culture medium alone) and controls (cells alone) were prepared at the same time. Three replicates were made for each measurement. 

### 2.6. Mediator Release Assay from KU812 Cells

KU812 cells (1.0 × 10^7^ cells per mL) were incubated with human serum at a final dilution of 1:10 at 37 °C in 5% CO_2_ for 24 h in 96-well plates. Sensitized cells were stimulated with 10 μL/well of ω-5 gliadin-derived peptides (150 μg/mL) at 37 °C in 5% CO_2_ for 4 h. The concentrations of ω-5 gliadin-derived peptides had no cytotoxic effect on KU812 cell. After centrifugation was performed at 120 g for 5 min, supernatants of cell cultures were collected. The contents of β-Hex and histamine were measured using commercial human enzyme-linked immunosorbent assay (ELISA) kits (Fusheng Industry Co. Shanghai, China), and interleukin 6 (IL-6) and TNF-α secretion were evaluated using commercial human ELISA kits (Neobioscience, Shenzhen, China) according to the manufacturer’s instructions. According to the result, the strongest allergenic peptide would be defined and used for subsequent experiments.

Subsequently, effects of flavonoids on ω-5 gliadin-derived peptide-induced degranulation of KU812 cells were evaluation. Briefly, after KU812 cells were incubated with human serum for 24 h, cells were pretreated with baicalein, luteolin, isorhamnetin, naringenin, positive control ketotifen fumarate and negative control sterile phosphate-buffered saline (PBS) for 0.5 h, followed by exposure to the strongest allergenic peptide of ω-5 gliadin-derived peptide (150 μg/mL) for 4 h. The concentrations of flavonoids and peptide had no cytotoxic effect on KU812 cell. Finally, the supernatant was collected, β-Hex, histamine, IL-6 and TNF-α content was measured.

### 2.7. Evaluation of the Effects of Four Flavonoids against the Reduction of Cell Viability Induced by ω-5 Gliadin-Derived Peptide

KU812 or Caco-2 cells were pretreated with different concentrations of baicalein, luteolin, isorhamnetin and naringenin for 3 h. The applied flavonoid concentrations had no cytotoxic effect on cell. Following, cells were exposed to the strongest allergenic peptide of ω-5 gliadin (500 μg/mL) for 24 h, and the concentration of peptide had obvious cytotoxicity on KU812 cell and Caco-2 cell. The viability tests were done using a Cell Counting Kit-8, as indicated by the producer.

### 2.8. Establishment of Caco-2 Cell Monolayer Model

Caco-2 cells were seeded on the apical compartment (AP) of 12-well Transwell plates at 2 × 10^5^ cells/mL in 0.5 mL DMEM complete medium. 1.5 mL DMEM complete medium was added into basolateral (BL) compartments. The basolateral medium was replaced every other day. From the tenth day of culture, the integrity of the monolayer was evaluated by measuring the transepithelial electrical resistance (TEER) using an Millicell ERS-2 volt ohm meter (Millipore, Bedford, MA, USA), and then measured every two days, Caco-2 cells were incubated for 18–21 days until the TEER > 800 Ω/cm^2^ [37]. The Caco-2 cell monolayer was shaped to differentiate and form tight junctions (TJ). 

### 2.9. Effects of Flavonoids on Secretion of IL-6 and IL-8 of Caco-2 Cells Induced by ω-5 Gliadin-Derived Peptide

The differentiated Caco-2 monolayers grown in Transwell plates were allocated into four groups: I, 0.5 mL 1% FBS DMEM medium was added to the apical compartment of the cell monolayer (blank), II. The cell monolayer was exposed to the screened strongest allergenic peptide of ω-5 gliadin (150 μg/mL) for 24 h followed by addition of 0.5 mL DMEM (negative control). III, Caco-2 monolayer was pretreated with 0.5 mL of 10 nM dexamethasone for 3 h, then was exposed to the screened ω-5 gliadin-derived peptide (150 μg/mL) for 24 h (positive control). IV, Caco-2 monolayer was preincubated with 0.5 mL of flavonoids for 3 h, and treated by the screened ω-5 gliadin-derived peptide (150 μg/mL) for 24 h (experimental groups). The experimental groups were subdivided into subgroups according to type and concentration of flavonoids.

After 24 h, the supernatant of the apical compartment of each well was collected. The supernatant was used for determination of IL-6 and IL-8 concentration by using commercial human ELISA kit (Neobioscience, Shenzhen, China) according to the manufacturer’s instructions.

### 2.10. Effects of Flavonoids on Tight Junction Damage of Caco-2 Cells Induced by ω-5 Gliadin-Derived Peptide

Release of zonulin and expression of tight junction proteins of Caco-2 cells were determined to evaluate the barrier integrity. Caco-2 monolayers were designated to four groups as mentioned above (Section 2.9). The supernatant of the apical compartment of Caco-2 monolayer was collected for the determination of the release of zonulin by commercial human zonulin ELISA kit (Fusheng Industry Co. Ltd., Shanghai, China) according to the manufacturer’s instructions. Followingly, caco-2 cells were lysed using Western and IP cell lysate (Beyotime Biotechnology, Shanghai, China) for 5 min, then centrifuged at 10,000× *g* for 5 min at 4 °C, and collect the supernatant for the determination of tight junction proteins Occludin and ZO-1. Total protein contents were quantified using the bicinchoninic acid assay (Beyotime Biotechnology, Shanghai, China) according to the manufacturer’s instructions. Cell protein (15 μg) was loaded onto SDS polyacrylamide gel (4% stacking gel; 8% separating gel) and separated by electrophoresis for 0.5 h at 12 mA and 2 h at 24 mA. Next, the separated proteins were transferred onto the nitrocellulose filter (NC) membrane in ice bath for 2.75 h at 100 V, 300 mA. The membranes were blocked with 5% skim milk in Tris-buffered saline containing 0.1% Tween-20 (TBST) for 1 h at 37 °C. After blocking, the membranes were incubated overnight at 4 °C with antibodies against occludin, ZO-1 and β-actin (Thermo Fisher Scientific, Waltham, MA, USA), followed by washing three times with TBST and incubation for 1 h at 37 °C with a horseradish peroxidase-conjugated goat anti-rabbit IgG antibody (Sigma-Aldrich, St.Louis, MO, USA). Reactive bands were detected using an enhanced chemiluminescence reagent kit (Sangon Biotech, Shanghai, China) and gel imaging system (Thermo Fisher Scientific, Waltham, MA, USA). Band intensity was quantified by GS-800 Calibrated Imaging Densitometer (Bio-Rad, Hercules, CA, USA), and the protein contents were normalized to β-actin.

### 2.11. Statistical Analysis

The GraphPad Prism 9 software was used for statistical analysis, and express the data of repeated experiments in the form of mean ± SDs. The significant differences between groups were evaluated by One-way ANOVA and Tukey’s multiple comparison test. When *p* < 0.05, it is considered that there is a significant difference between the groups compared with each other.

## 3. Results

### 3.1. Identification of Gastrointestinal Digestion Products of Gliadin

After gliadin was digested using an infant vitro model of gastroduodenal digestion, the digestion products are detected by Tricine-SDS-PAGE (Figure S1) and RP-HPLC (Figure S2), respectively. Gliadin sample in the study includes α/β-gliadin and γ-gliadin (31–45 kDa), ω-gliadin(~60 KDa) and some α-amylase inhibitor (~15 KDa). After simulated gastric digestion, the bands corresponding to all these proteins became weak. When the simulated intestinal digestion was over, the gliadin was almost completely digested, and no obvious bands were seen (Figure S1). 

The results of RP-HPLC were consistent with the results of Tricine-SDS-PAGE. After simulated gastric digestion in vitro, gliadin was not completely digested. After intestinal digestion, the characteristic peaks of various types of gliadin disappeared and small chromatographic peaks appeared, indicating that ω-, α/β-, γ-gliadins were almost completely digested in the intestinal digestion stage, and became smaller molecular weight peptides (Figure S2).

### 3.2. Selected and Synthesized of ω-5 Gliadin-Derived Peptide

The end products of gastrointestinal digestion were analyzed by LC-MS, and the results showed that after gliadin was digested in the simulated gastrointestinal tract in vitro, there were 93 digested peptides of ω-5 gliadin, and the molecular weights were mainly distributed in 3–4 Kda (Table S2). Combining the main allergen epitopes in ω-5 gliadin (Table 1) and the results of LC-MS, four peptides were screened. The sequences of these four peptides contained multiple ω-5 gliadin allergen epitopes (Table 2).

### 3.3. Effect of Peptides and Flavonoids on KU812 Cell Viability

The concentrations of four peptides and four flavonoids without affecting the proliferation activity of KU812 cells were determined. The results showed the cell viability was still greater than 95% when 150 μg/mL of peptides P1, P2, P3 and P4 was incubated with cells, respectively, that indicating the sample has no effect on cell proliferation [38] (Figure S3), then 150 μg/mL was selected as the working concentration of each peptide for further experiments. However, 500 μg/mL of peptide P4 decreased cell viability significantly, and the cell viability was 80.0 % (Figure S3). Flavonoid baicalein at concentrations below 30 μM, luteolin and isorhamnetin at concentrations below 5 μM, and naringenin at concentrations below 50 μM, all had no significant effect on the viability of KU812 cells. The cell viability was greater than 95%, and positive control ketotifen fumarate (1 μM~100 μM) also had no significant effect on KU812 cell viability (Figure S4). The results also suggested that luteolin and isorhamnetin cytotoxicity were greater than that of baicalein and naringenin.

### 3.4. Selected ω-5 Gliadin-Derived Peptides with Strong Allergenicity Based on KU812 Cell Degranulation Model

**Figure 1:**
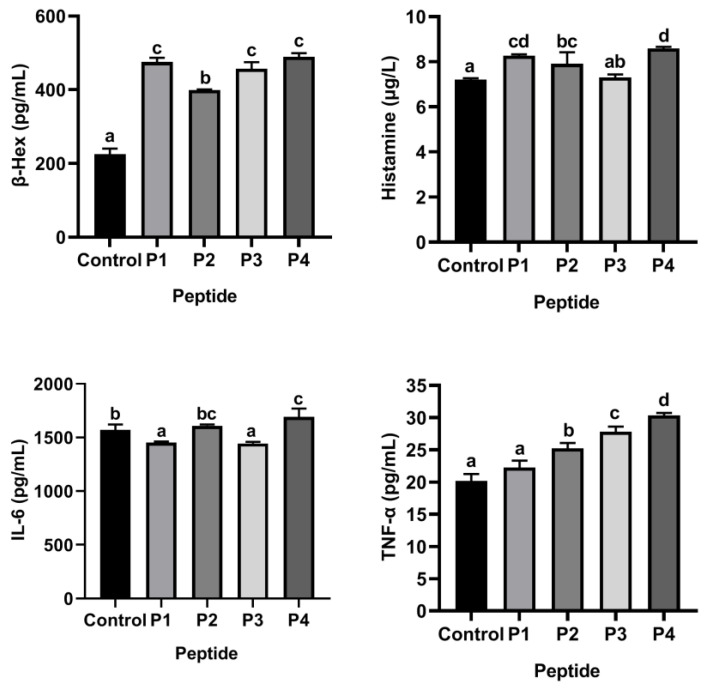
β-Hex, histamine, IL-6 and TNF-α release of KU812 cells in the presence of ω-5 gliadin-derived peptides (150 μg/mL). Note: Different letters indicate significant differences between groups (*p* < 0.05).

ω-5 gliadin-derived peptides with strong allergenicity were screened based on KU812 cell degranulation model. Compared with control group without synthetic peptide treatment, only 150 μg/mL of peptide P4 promoted β-Hex, histamine and TNF-α release from KU812 cells at the same time (Figure 1), indicating that ω-5 gliadin-derived peptide P4 had the strongest ability to induce degranulation of KU812 cell among the four ω-5 gliadin-derived peptides. It can be considered that the ω-5 gliadin-derived peptide P4 (AA_253–279_, PQQPQQFPQQQQFPQQQSPQQQQFPQQ) is the strongest allergenic peptide and is used for subsequent experiments.

### 3.5. Flavonoids Isorhamnetin, Naringenin, Luteolin and Baicalein Attenuated Cytotoxicity of ω-5 Gliadin-Derived Peptide P4 to KU812 Cells

**Figure 2:**
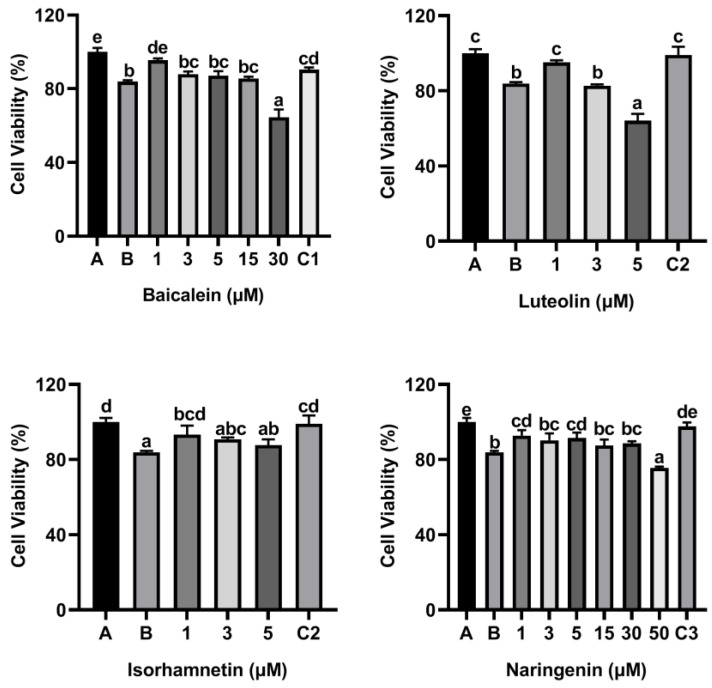
Effects of four flavonoids on the viability of KU812 cells induced by ω-5 gliadin-derived peptide P4. A (blank group): only KU812 cells; B (control group): 500 μg/mL ω-5 gliadin-derived peptide P4+KU812 cells; C1 (positive control group): 30 μM ketotifen fumarate + KU812 cells; C2 (positive control group): 5 μM ketotifen fumarate + KU812 cells; C3 (positive control group): 50 μM ketotifen fumarate + KU812 cells. Note: different letters indicate significant differences between groups (*p* < 0.05).

As shown Figure 2, the cell viability was decreased to 83.82% when KU812 cells were exposed to 500 μg/mL ω-5 gliadin-derived peptide P4. Treatment with baicalein (1 μM), luteolin (1 μM), isorhamnetin (1 μM), and naringenin (1 μM, 5 μM) significantly inhibited the decrease of KU812 cell viability induced by peptide P4 (*p* < 0.05). However, 30 μM baicalein, 5 μM luteolin and 50 μM naringenin increased cytotoxicity of ω-5 gliadin-derived peptide P4 to KU812 Cells. These results indicated that low dose baicalein, luteolin, isorhamnetin and naringenin could inhibit the decline of KU812 cell viability induced by peptide P4 and had cytoprotective effects on KU812 cells.

### 3.6. Flavonoids Inhibited the Release of Inflammatory Mediators from KU812 Cells Induced by ω-5 Gliadin-Derived Peptide P4

**Figure 3:**
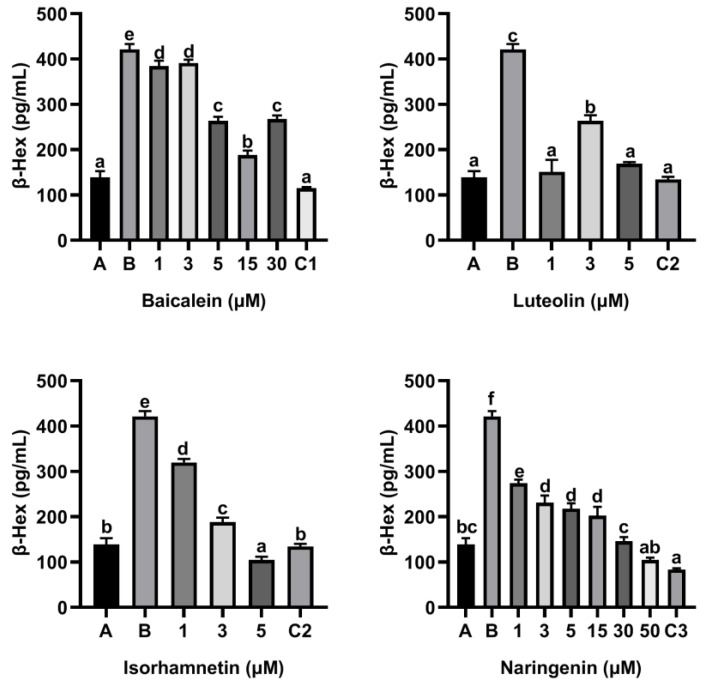
Effect of four flavonoids on the release of β-Hex from KU812 cells induced by ω-5 gliadin-derived peptide P4. A (blank group): Only KU812 cells; B (control group): 150 μg/mL ω-5 gliadin-derived peptide P4+KU812 cells; C1 (positive control group): 30 μM ketotifen fumarate + KU812 cells; C2 (positive control group): 5 μM ketotifen fumarate + KU812 cells; C3 (positive control group): 50 μM ketotifen fumarate + KU812 cells. Note: different letters indicate significant differences between groups (*p* < 0.05).

To access the effect of flavonoids on the release of inflammatory mediators, KU812 cells were cultured with flavonoids baicalin, luteolin, isorhamnetin and naringenin for 0.5 h, respectively, and then stimulated with 150 µg/mL ω-5 gliadin-derived peptide P4 for 4 h. As shown in Figure 3, compared with the control group only treated with peptide P4 (150 μg/mL), all concentrations of baicalein, luteolin, isorhamnetin and naringenin reduced the release of β-Hex induced by peptide P4 (*p* < 0.05), and the effect of isorhamnetin at a concentration of 5 µM was greater than that of 5 µM ketotifen fumarate (*p* < 0.05). In addition, the β-Hex release was suppressed by isorhamnetin with dose dependent manner (Figure 3). 

**Figure 4:**
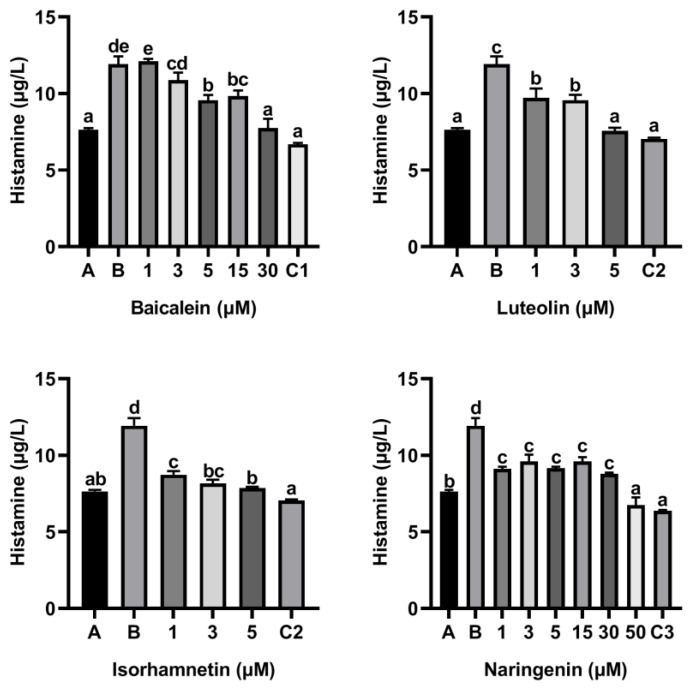
Effect of four flavonoids on the release of histamine from KU812 cells induced by ω-5 gliadin-derived peptide P4. A (blank group): only KU812 cells; B (control group): 150 μg/mL ω-5 gliadin-derived peptide P4+KU812 cells; C1 (positive control group): 30 μM ketotifen fumarate + KU812 cells; C2 (positive control group): 5 μM ketotifen fumarate + KU812 cells; C3 (positive control group): 50 μM ketotifen fumarate + KU812 cells. Note: different letters indicate significant differences between groups (*p* < 0.05).

Moreover, flavonoids baicalein, luteolin, isorhamnetin and naringenin all inhibited the release of histamine from KU812 cells induced by peptide P4, and the release of histamine from KU812 cells decreased gradually with the increasing of flavonoid concentration (Figure 4). The effect of 30 μM baicalein, 5 μM luteolin and 50 μM naringenin in inhibiting histamine release was not significantly different from that of ketotifen fumarate at the same concentration (Figure 4).

**Figure 5:**
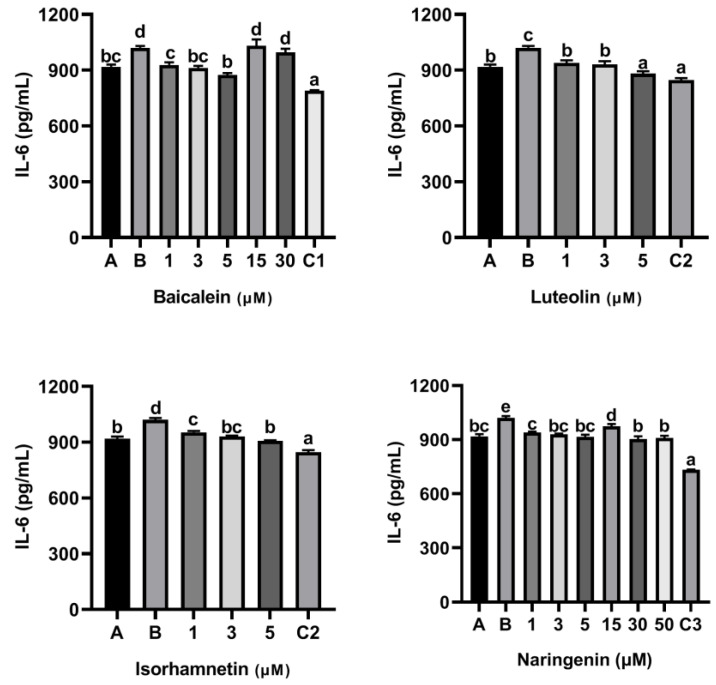
Effect of four flavonoids on the release of IL-6 from KU812 cells induced by ω-5 gliadin-derived peptide P4. A (blank group): only KU812 cells; B (control group): 150 μg/mL ω-5 gliadin-derived peptide P4+KU812 cells; C1 (positive control group): 30 μM ketotifen fumarate + KU812 cells; C2 (positive control group): 5 μM ketotifen fumarate + KU812 cells; C3 (positive control group): 50 μM ketotifen fumarate + KU812 cells. Note: different letters indicate significant differences between groups (*p* < 0.05).

As shown Figure 5, compared with the control group only treated with peptide P4 (150 μg/mL), baicalein, luteolin, isorhamnetin and naringenin all reduced the release of IL-6 induced by peptide P4. Notability, the inhibitory effect of baicalein on release of IL-6 induced by peptide P4 did not increase with increasing concentration, and when the acting concentration of baicalein increased to 15 µM and 30 µM, the inhibitory effect disappeared. 

**Figure 6:**
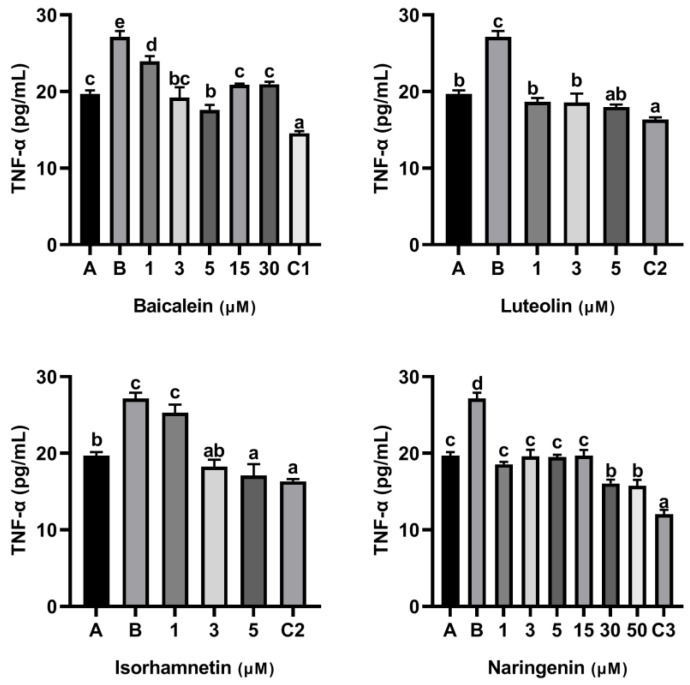
Effect of four flavonoids on the release of TNF-α from KU812 cells induced by ω-5 gliadin-derived peptide P4. A (blank group): only KU812 cells; B (control group): 150 μg/mL ω-5 gliadin-derived peptide P4+KU812 cells; C1 (positive control group): 30 μM ketotifen fumarate + KU812 cells; C2 (positive control group): 5 μM ketotifen fumarate + KU812 cells; C3 (positive control group): 50 μM ketotifen fumarate + KU812 cells. Note: different letters indicate significant differences between groups (*p* < 0.05).

Furthermore, KU812 cells also released TNF-α when the cells were stimulated with ω-5 gliadin-derived peptide P4 (Figure 6). Compared with the control group treated with peptide P4 (150 μg/mL), baicalein, luteolin, isorhamnetin and naringenin all reduced the release of TNF-α induced by peptide P4. In addition, similar to the inhibitory effect of baicalein on release of IL-6, the inhibitory effect of baicalein on release of TNF-α induced by peptide P4 did not increase with increasing concentration too, and when the acting concentration of baicalein increased to 15 µM and 30 µM, the inhibitory effect decreased (Figure 6). 

**Figure 7:**
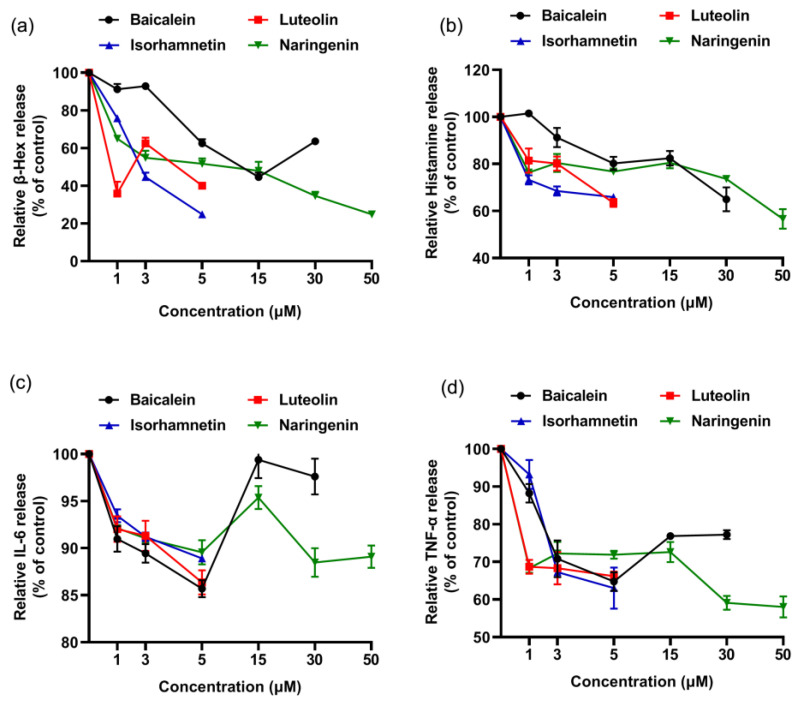
Dose-effect relationship of four flavonoids inhibiting mediator release from KU812 cells induced by ω-5 gliadin-derived peptide P4. (**a**) Dose-effect relationship of flavonoids for inhibition β-Hex release; (**b**) dose-effect relationship of flavonoids for inhibition histamine release; (**c**) dose-effect relationship of flavonoids for inhibition IL-6 release; (**d**) dose-effect relationship of flavonoids for inhibition TNF-α release.

In addition, we drew dose- effect curve, and compared the anti-degranulation activity and the anti-inflamatory efficacy of the four flavonoids (Figure 7). Except baicalein, the anti-inflamatory efficacy of luteolin, isorhamnetin and naringenin increased with increasing concentration. At a concentration of 5 µM, we found the anti-degranulation activity of isorhamnetin and luteolin was greater than that of naringenin and baicalein. 

The above results demonstrated that the release of inflammatory mediator from activated KU812 cells was down-regulated by baicalein, luteolin, isorhamnetin and naringenin, and indicated that the four flavonoids inhibit degranulation of basophils stimulated by ω-5 gliadin-derived peptide, and have a certain anti-allergic effect.

### 3.7. Effect of Peptide P4 and Flavonoids on Caco-2 Cell Viability

The concentrations of peptide P4 and four flavonoids without affecting the proliferation activity of Caco-2 cells were determined. The results showed peptide P4 (5~150 μg/mL) was incubated with Caco-2 cells for 24 h, and the cell viability was greater than 95%; while 300 μg/mL and 500 μg/mL peptide P4 acted on Caco-2 cells, the average cell viability was 93.82% and 86.55%, respectively, and the cell viability was less than 95% (Figure S5), so these two concentrations will affect the proliferation of Caco-2 cells. Therefore, 150 μg/mL was selected as the working concentration of peptide P4 for further experiments.

Flavonoids baicalein and naringenin at concentrations below 30 μM, luteolin at concentrations below 15 μM, and isorhamnetin at concentrations below 50 μM had no significant effect on the viability of Caco-2 cells, and the cell viability was greater than 95% (Figure S6). In addition, according to our previous study, 10 nM dexamethasone acts on Caco-2 cells, and its cell viability is greater than 95%, and has no effect on cell proliferation.

### 3.8. Flavonoids Isorhamnetin, Naringenin, Luteolin and Baicalein Attenuated Peptide P4-Induced Cytotoxicity in Caco-2 Cells

**Figure 8:**
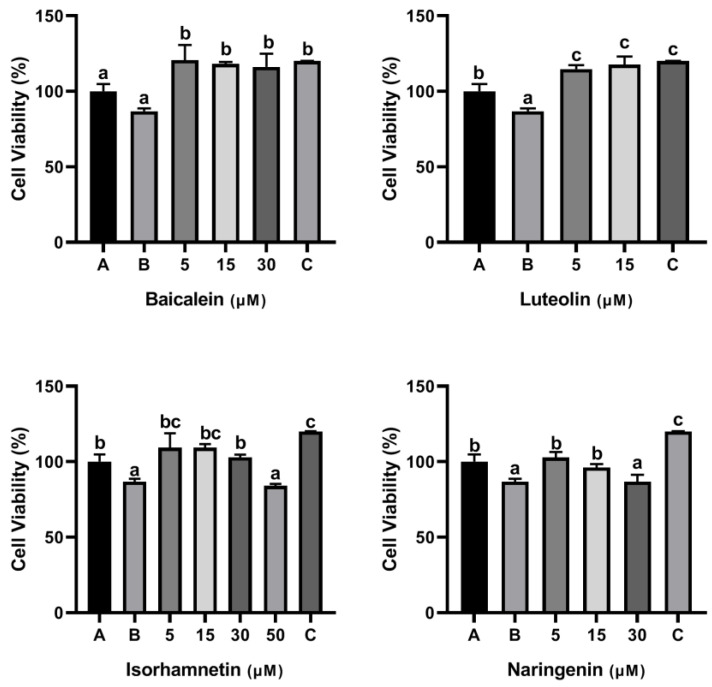
Effects of four flavonoids on the viability of Caco-2 cells induced by ω-5 gliadin-derived peptide P4. A (blank group): only Caco-2 cells; B (control group): 500 μg/mL ω-5 gliadin-derived peptide P4+Caco-2 cells; C (positive control group): 10 nM Dexamethasone+Caco-2 cells. Note: different letters indicate significant differences between groups (*p* < 0.05).

As shown Figure 8, the cell viability was decreased to 86.85% when Caco-2 cells were exposed to 500 μg/mL ω-5 gliadin-derived peptide P4. Treatment with baicalein, luteolin, isorhamnetin and naringenin significantly inhibited the decrease of Caco-2 cell viability induced by peptide P4, except 50 μM isorhamnetin and 30 μM naringenin (Figure 8). These results indicated that baicalein, luteolin, isorhamnetin and naringenin could inhibit the decline of Caco-2 cell viability induced by peptide P4 and had cytoprotective effects on Caco-2 cells.

### 3.9. Flavonoids Inhibited the Release of IL-6 and IL-8 from Caco-2 Cell Monolayers Induced by ω-5 Gliadin-Derived Peptide P4

To investigate the regulatory effects of four flavonoids on inflammatory response of Caco-2 cells induced by ω-5 gliadin-derived peptides, we established a Caco-2 intestinal epithelial cell monolayer model and pre-incubated with four flavonoids for 3 h. Then, 150 µg/mL of ω-5 gliadin-derived peptide P4 with strong allergenicity was added for 24 h, and the contents of inflammatory factors IL-6 and IL-8 were detected by ELISA.

As shown in Figure S7, the TEER of the Caco-2 cell monolayer cultured to 16–21 days was higher than 800 Ω/cm^2^, the tight junctions were considered complete and the monolayer mode was established successfully [37].

**Figure 9:**
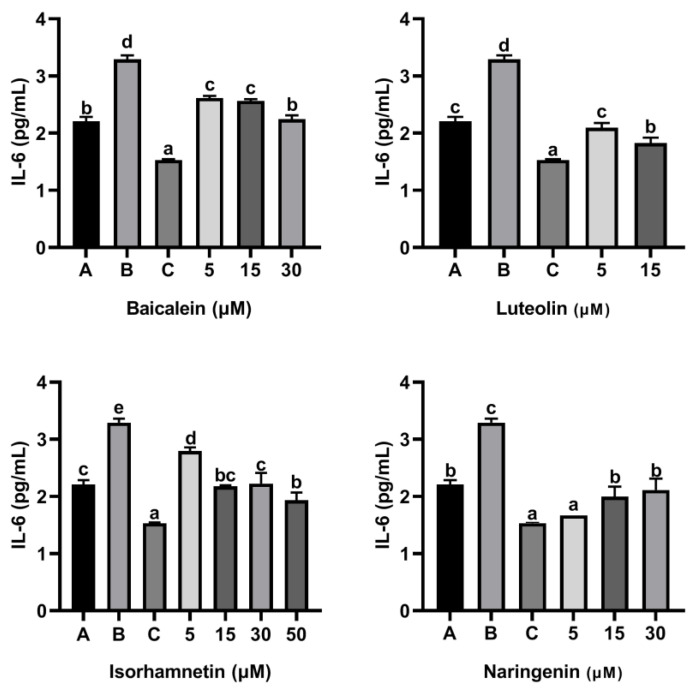
Effect of four flavonoids on the release of IL-6 from Caco-2 cells induced by ω-5 gliadin-derived peptide P4. A (blank group): only Caco-2 cells; B (control group): 150 μg/mL ω-5 gliadin-derived peptide P4+Caco-2 cells; C (positive control group): 10 nM Dexamethasone+Caco-2 cells. Note: different letters indicate significant differences between groups (*p* < 0.05).

**Figure 10:**
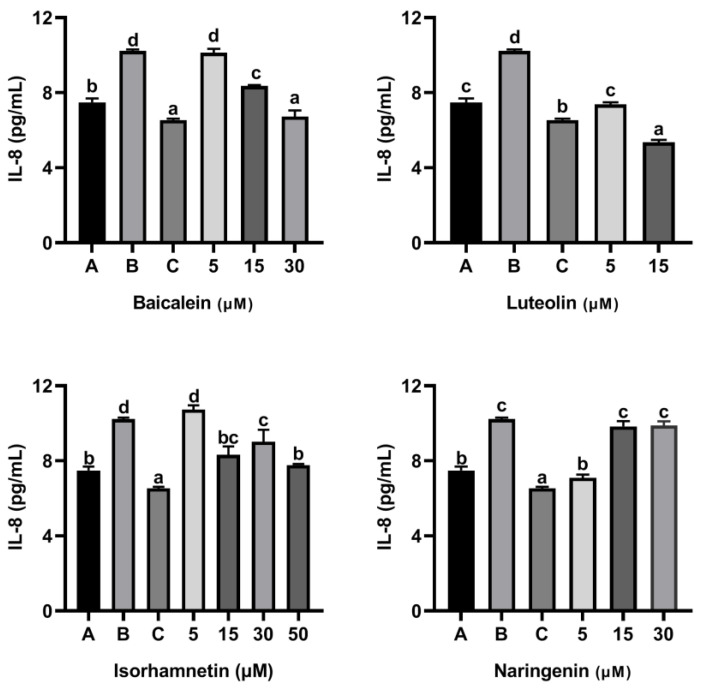
Effect of four flavonoids on the release of IL-8 from Caco-2 cells induced by ω-5 gliadin-derived peptide P4. A (blank group): only Caco-2 cells; B (control group): 150 μg/mL ω-5 gliadin-derived peptide P4+Caco-2 cells; C (positive control group): 10 nM Dexamethasone+Caco-2 cells. Note: different letters indicate significant differences between groups (*p* < 0.05).

As shown in Figure 9 and Figure 10, ω-5 gliadin-derived peptide P4 significantly promoted the release of the inflammatory factor IL-6 and IL-8 in Caco-2 cells. Compared with the control group treated with peptide P4, baicalein, luteolin, isorhamnetin and naringenin significantly inhibited the release of IL-6 from Caco-2 cells induced by ω-5 gliadin-derived peptide P4 (*p* < 0.05). Baicalein (30 μM), luteolin (5 μM, 15μM), isorhamnetin (15 μM, 30 μM, 50 μM) and naringenin (5 μM, 15 μM, 30 μM) suppressed the secretion of IL-6 to normal levels, even below normal levels. Moreover, different concentration of the tested flavonoids also significantly inhibited the release of IL-8 from Caco-2 cells, except 5 μM baicalein, 5 μM isorhamnetin, 15 μM and 30 μM naringenin. The inhibitory effects of baicalein (30 μM) and luteolin (15 μM) were similar to that of positive control dexamethasone (10 nM).

**Figure 11:**
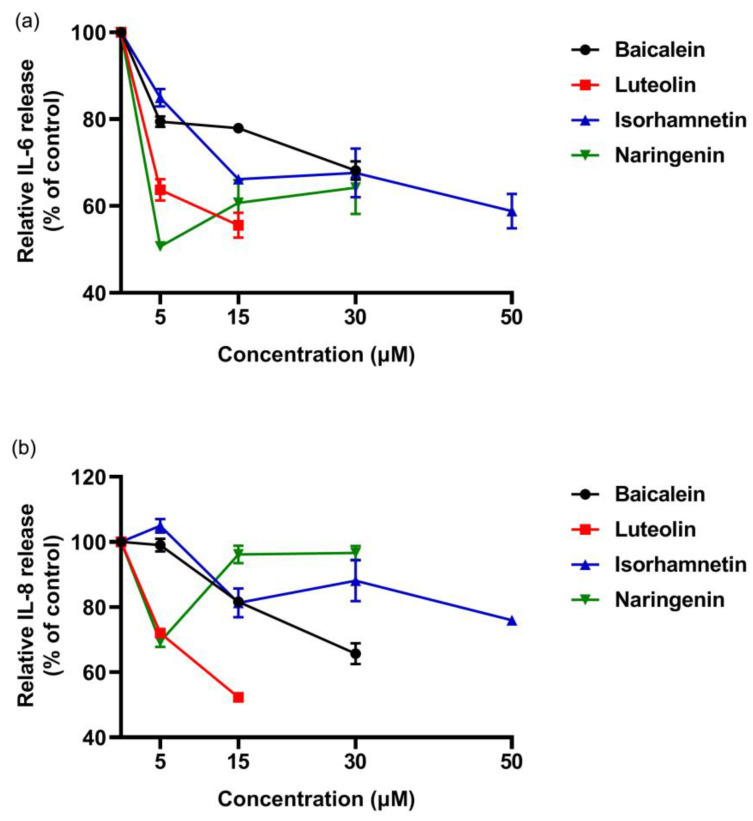
Dose-effect relationship of four flavonoids inhibiting IL-6 and IL-8 release from Caco-2 cells induced by ω-5 gliadin-derived peptide P4. (**a**) Dose-effect relationship of flavonoids for inhibition IL-6 release; (**b**) dose-effect relationship of flavonoids for inhibition IL-8 release.

These results indicated that the four flavonoids reduced the inflammation reaction of Caco-2 cells caused by ω-5 gliadin-derived peptide. Notably, baicalein, luteolin and isorhamnetin gradually decreased the secretion of IL-6 and IL-8 with the increase of concentration, while naringenin treatment showed the opposite effect (Figure 11). The inhibitory effect decreased when the acting concentration of naringenin increased from 5 µM to 15 µM and 30 µM, even the inhibitory effect to IL-8 release disappeared. In addition, the inhibitory action of naringenin and luteolin on the release of IL-6 and IL-8 from Caco-2 cells was greater than that of baicalein and isorhamnetin at a concentration of 5 µM, which was different from the order of the anti-degranulation effect of the four flavonoids.

### 3.10. Flavonoids Improved Intestinal Epithelial Tight Junction Damage Induced by ω-5 Gliadin-Derived Peptide

**Figure 12:**
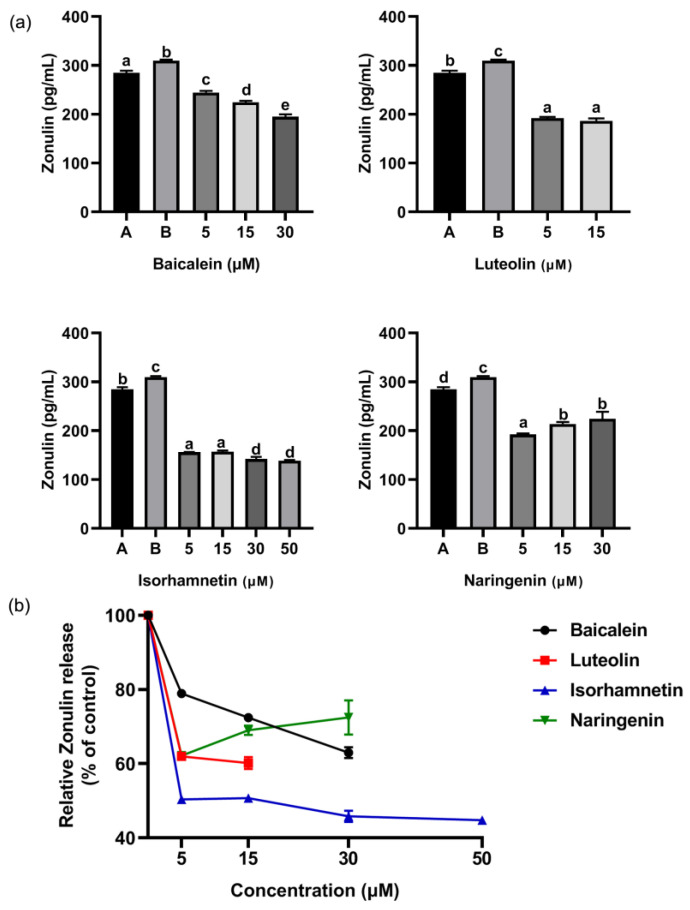
(**a**) Effect of four flavonoids on the release of Zonulin from Caco-2 cells induced by ω-5 gliadin-derived peptide P4. A (blank group): only Caco-2 cells; B (control group): 150 μg/mL ω-5 gliadin-derived peptide P4+Caco-2 cells; note: different letters indicate significant differences between groups (*p* < 0.05); (**b**) dose-effect relationship of four flavonoids of inhibition zonulin release.

**Figure 13:**
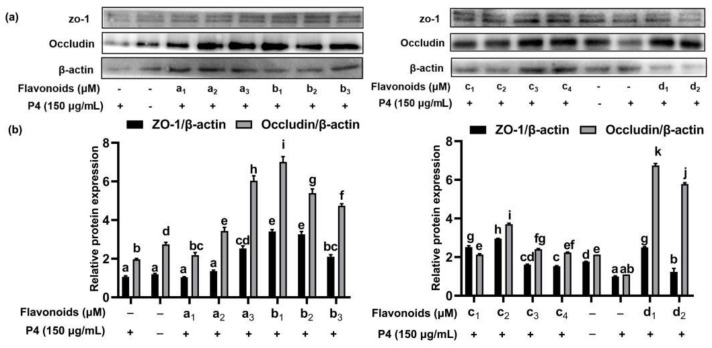
Effect of four flavonoids pretreatment on expression of occludin and ZO-1 protein. (**a**) Representative immunoblots of the indicated proteins are shown; (**b**) relative protein expression of occludin and ZO-1; a_1_: 5 μM baicalein; a_2_: 15 μM baicalein; a_3_: 30 μM baicalein; b_1_: 5 μM naringenin; b_2_:15 μM naringenin; b_3_: 30 μM naringenin; c_1_: 5 μM isorhamnetin; c_2_: 15 μM isorhamnetin; c_3_: 30 μM isorhamnetin; c_4_: 50 μM isorhamnetin; d_1_: 5 μM luteolin; d_2_: 15 μM luteolin; +: cells were treated with peptide P4; -: cells weren’t treated with flavonoids or peptide P4. Note: different letters indicate significant differences between groups (*p* < 0.05).

ω-5 gliadin-derived peptide P4 promoted the release of zonulin (Figure 12), and reduced the expression of tight junction proteins occludin and ZO-1 in Caco-2 cell monolayers (Figure 13), led to tight junction damage. Treatment with baicalein, luteolin, isorhamnetin and naringenin significantly reduced the release of zonulin induced by peptide P4, but the effect of naringenin decreased with increasing concentration (Figure 12). The effect of isorhamnetin was strongest among the four flavonoids (Figure 12). In addition, the four flavonoids significantly increased the expression of tight junction proteins occludin and ZO-1 in Caco-2 cell monolayers (Figure 13). With the increase of the concentration of baicalein and luteolin, the expression of tight junction proteins occludin and ZO-1 increased, while the effect of naringenin was opposite (Figure 13). 

These results suggested that flavonoids repaired intestinal epithelial tight junction damage induced by ω-5 gliadin-derived peptide, may improve intestinal barrier function. In addition, we found whether inhibiting the release of inflammatory mediators or enhancing the expression of tight junction proteins in Caco-2 cell, the effect of naringenin decreased with the increase of concentration, with the optimal concentration at 5 μM. The results were not consistent with the results of anti-degranulation of naringenin.

## 4. Discussion

In the present study, ω-5 gliadin peptide, main allergen of pediatric wheat food allergy, was used to induce KU812 cell degranulation and intestinal epithelial tight junction damage in vitro. Gliadin contains a high content of proline amino acid, rendering it highly resistant to degradation in the gastrointestinal tract [39]. After gliadin was digested using an infant vitro model of gastroduodenal digestion, 93 digested peptides from ω-5 gliadin were identified by LC-MS, and the majority of these peptides contained multiple epitopes. Based on the appropriate length of peptide and the number of allergenic epitope included in peptide, four peptides obtained by digestion of ω-5 gliadin were screened out and estimated allergenicity by KU812 cell degranulation model. KU812 cells are human basophils that express high-affinity IgE receptors on their surface. Basophils and mast cells are the main effector cells involved in type I hypersensitivity. The activation of basophils and mast cells by antigen results in the degranulation of pre-stored granules (e.g., histamine and β-Hex) and the release of newly synthesized inflammatory cytokines and chemokines (degranulation-independent manner), which are often used in food allergen allergenicity studies [40,41]. Many stimuli have been proposed to activate basophils and mast cell degranulation or cytokine production. In the study, compared with the other three peptides, the ω-5 gliadin-derived peptide P4 (AA_253–279_, PQQPQQFPQQQQFPQQQSPQQQQFPQQ) was the strongest allergenic peptide and could significantly promote the degranulation of KU812 cells and the release of histamine, β-Hex, IL-6 and TNF-α. Then, we studied the effects of baicalein, luteolin, isorhamnetin and naringenin four flavonoids, on KU812 cell degranulation induced by ω-5 gliadin-derived peptide P4. It was observed that before the four flavonoids significantly attenuated peptide P4 induced cytotoxicity, they inhibited the release of β-Hex, histamine, IL-6 and TNF-α of KU812 cells stimulated by peptide P4, indicating these flavonoids have anti-degranulating activity. The results were consistent with the previous reports. Degranulation inhibitory activities of luteolin, baicalein and naringenin have been described in several reports [26,28,31,42]. Luteolin was reported to show degranulation inhibitory activities in antigen-stimulated rat basophil leukemia RBL-2H3 cells (IC_50_ 5.8 µM) [42], and suppressed Substance P (SP) or IgE/anti-IgE–stimulated β-Hex, histamine and TNF-α release from LAD2 mast cells [28]. Baicalein (1.8 to 30 μM) significantly inhibited release of IL-6 and IL-8 from IL-1β-activated human mast cells in a dose-dependent manner via regulation of the NF-ḳB pathway with the optimal inhibition concentration at 30 μM [26]. Naringenin (50 µM, 200 µM) and baicalein (25 µM, 50 µM) inhibited the release of β-Hex from RBL-2H3 cells induced by human serum albumin and suppressed the phosphorylation of Akt [31]. Compared to the previous reports, our study directly demonstrated the inhibitory effect of these three flavonoids on wheat antigen-stimulated degranulation. In addition, there has been no report on the suppression of degranulation by isorhamnetin. Our study found isorhamnetin effectively inhibited degranulation of KU812 cell induced by ω-5 gliadin-derived peptide P4, and the effect of flavonol isorhamnetin was greater than that of flavones baicalein and flavanone naringenin. 

Intestinal barrier function is responsible for intestinal health and disease, which is controlled by tight junctions between adjacent epithelial cells. Tight junction, a multiprotein complex consisting of transmembrane proteins (occludin, claudins, junction adhesion molecule and tricellulin) and cytosolic scaffold proteins zonula occludins (ZOs), selectively modulated the paracellular permeability and intestinal barrier function [43]. The impairment of barrier function is implicated in intestinal pathogenesis, including inflammatory bowel diseases (IBD), alcoholic liver diseases, celiac disease and food allergies [43]. Therefore, the protection of intestinal barrier integrity could afford preventive and therapeutic approaches for these diseases. Caco-2 cells are human colon adenocarcinoma cells, and the Caco-2 intestinal epithelial cell monolayer model has been widely used to evaluate the effect of potential inhibitors on inflammation-induced intestinal barrier dysfunction [44,45,46]. Previous studies have shown that bacterial infection and gluten can stimulate intestinal mucosal secretion of zonulin (a putative regulator of tight junction permeability), damage intestinal tight junction proteins, and increase intestinal permeability [47]. In the study, the ω-5 gliadin-derived peptide P4 significantly promoted the release of IL-6, IL-8 and zonulin in Caco-2 cell monolayer, and suppressed the expression of tight junction proteins, led to the barrier damage in Caco-2 intestinal epithelial monolayers, which are consistent with previous studies. The treatment of baicalein, luteolin, isorhamnetin and naringenin significantly improved the decline of Caco-2 cell viability induced by peptide P4, inhibited the release of IL-6, IL-8 and zonulin from caco-2 cell monolayers induced by ω-5 gliadin-derived peptide P4, increased the expression of tight junction proteins occludin and ZO-1, and repaired intestinal epithelial tight junction damage induced by ω-5 gliadin-derived peptide P4. The results were consistent with previous reports. Luteolin was reported to inhibit the production of inflammatory cytokines TNF-α, IL-6 and IL-1β, increase the expression of tight junction proteins ZO-1, occludin and claudin-1, reduce BDE-209 induced Caco-2 intestinal epithelial barrier damage [48]; repair the ethanol-induced Caco-2 cell monolayer barrier disrupted through regulating MAPK/NF-κB/MLCK-mediated tight junction pathways and Keap1/Nrf2-ARE-mediated antioxidant responses pathways [49]. In addition, in vivo experiments in mice also identified that luteolin restored the expression of ZO-1, occludin and claudin-1 in the intestine and alleviated increased intestinal permeability caused by a high-fat diet, thus enhancing the intestinal barrier function [50]. Naringenin enhanced the integrity of the intestinal barrier by increasing the expression of occludin and claudin-4 in Caco-2 cells [51]; prevented hyperglycemic-induced oxidative stress and membrane leakage in Caco-2 cells [52]; and supplementing naringenin ameliorated the DSS-induced colitis through protection of the tight junction barrier and the anti-inflammatory effect [53]. Jang H et. al. showed that baicalein significantly ameliorated radiation-induced intestinal injury by decreasing leukocyte filtration, inhibiting intestinal inflammation response and improving intestinal barrier dysfunction [54]. But there has been no report on the effect of isorhamnetin on intestinal epithelial barrier function. Our study found isorhamnetin also repaired intestinal epithelial tight junction damage induced by ω-5 gliadin-derived peptide, promoted epithelial barrier function, and the effect of flavonol isorhamnetin was strongest among the four flavonoids. There were evidences that a high-fat diet damaged gut barrier function through disrupting tight junctions, modulating the intestinal mucus composition and gut microbial community structure, promoting an inflammatory response, which promotes food allergy, celiac disease and inflammatory bowel disease [55,56]. The improvement of intestinal barrier function by flavonoids means flavonoid rich diet maybe be of benefit for human health.

In the study, it is worth noting the dose-effect curve of baicalein in KU812 degranulation was U-shaped, and corresponding to different effect indicators, the optimal concentration of baicalein were different, which was not consistent with a previous report [26]. Moreover, it was found that the dose-effect relationship of naringenin was negative according to the results of Caco-2 cell experiment. These existing problems will be further studied. In addition, we only observed the effect of three different subclass flavonoids on inhibiting degranulation and enhancing intestinal barrier, but did not investigate the structure-activity relationship in the study. Previous studies demonstrated that flavonoids, including the tested flavonoids naringenin [57] and luteolin [58] in the study, can inhibit the antigenicity of milk allergen β-lactoglobulin through non-covalent interactions, and the structure of flavonoids and antigenicity reduction of β-lactoglobulin are correlated [57]. Thus, it can be seen that the flavonoid-gliadin peptide P4 interaction maybe be responsible for the protective effect against allergenicity. The structure-activity relationship of the tested flavonoids needs further research in the future.

## 5. Conclusions

Selected flavonoids (baicalein, luteolin, isorhamnetin and naringenin) can significantly inhibit the degranulation of KU812 cells stimulated by ω-5 gliadin-derived peptide P4 and the release of proinflammatory cytokines; reduce ω-5 gliadin-derived peptide P4-induced inflammation of Caco-2 cells and enhance TJ protein levels. They might also be able to inhibit intestinal penetration of food allergens such as gliadin, which supports the potential pharmaceutical application of the four flavonoids treatment for wheat food allergy.

Notability, this is the first report, as far as we know, to identify that isorhamnetin can inhibit basophils degranulation and enhance intestinal barrier function. Further detailed studies on the anti-allergy mechanisms of isorhamnetin are now underway. 

## Figures and Tables

**Table 1:** Major allergen epitope in ω-5 gliadin [9,10,36].

Allergen	Biochemical Name	Molecular Weight (Kda)	Allergen Epitope
Tri a 19	ω-5 gliadin	49~55	QQX1PX2QQ (X1 = L, F, S, I, Y; X2 = Q, E, G)

**Table 2:** Amino acids sequences of allergenic epitope-bearing peptides of ω-5 gliadin obtained by digestion of gliadin.

Peptide	Sequence	the Number of Epitopes
P1: AA_374–404_	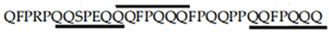	3
P2: AA_160–189_	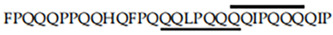	2
P3: AA_252–271_	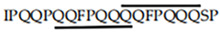	2
P4: AA_253–279_	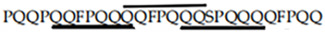	3

## Data Availability

Date is contained within the article and Supplementary Material.

## Supplementary Materials

The following supporting information can be downloaded at: https://www.mdpi.com/article/10.3390/foods11233857/s1, Table S1: Information of pediatric patients with wheat allergy; Table S2: Amino acids sequences, fragment labels, and masses of peptides of ω-5 gliadin obtained by digestion of gliadin; Figure S1: Tricine-SDS-PAGE of gastrointestinal digestion products of gliadin; Figure S2: RP-HPLC chromatogram of gastrointestinal digestion products of gliadin; Figure S3: Effect of ω-5 gliadin-derived peptides on the viability of KU812 cells; Figure S4: Effect of four flavonoids and ketotifen fumarate on the viability of KU812 cells; Figure S5: Effect of ω-5 gliadin-derived peptides P4 on the viability of Caco-2 cells; Figure S6: Effect of four flavonoids on the viability of Caco-2 cells; Figure S7: Determination of TEER of the Caco-2 cell monolayer.
