# Supplementary material for: In Vitro Effect of Flavonoids on Basophils Degranulation and Intestinal Epithelial Barrier Damage Induced by ω-5 Gliadin-Derived Peptide
Source: Foods. 2022 Nov 29;11(23):3857. doi: 10.3390/foods11233857 (PMC9741160; doi:10.3390/foods11233857)
Supplement: Supplementary file 1 [file foods-11-03857-s001.zip › foods-1918726-supplementary.pdf]

Table S1. Information of pediatric patients with wheat allergy

Patient no.	Sex	Age	Clinical symptoms
1	Male	6 year	Allergic rhinitis, atopic dermatitis
2	Male	3 year	Rhinitis, severe allergic reaction
3	Female	7 year	Rhinitis, Dermatitis
4	Female	8 year	Eczema
5	Male	3 year	Atopic dermatitis
6	Male	3 year	Hives
7	Male	4 year	Eczema
8	Male	3 year	Cough, Rhinitis, Eczema
9	Male	7 year	Allergic rhinitis, stomach pain
10	Male	2 year	Atopic dermatitis
11	Male	4 year	Hives
12	Male	6 year	Hives
13	Male	9 year	Asthma

Table S2: Amino acids sequences, fragment labels, and masses of peptides of ω-5 gliadin obtained by digestion of gliadin.

Pro	Squence	Fragment label	Mass （Da）
ω-5 gliadin	LSPRGKELHTPQEQFPQQQQFP QPQQFPQQQIPQQHQIPQ	23-62	4800.45
ω-5 gliadin	QQHQIPQQPQQFPQQQQFLQQQ QIPQQQIPQQHQIPQQP	56-94	4778.43

ω-5 gliadin	QQQQFPQQQQLTQQQFPRPQQS PEQQQFPQQQFPQQP	360-396	4562.16
ω-5 gliadin	QQFPRPQQSPEQQQFPQQQFPQ QPPQQFPQQQFPI	373-407	4291.08
ω-5 gliadin	QQSPEQQQFPQQQFPQQPPQQF PQQQFPIPYPPQ	379-412	4119.94
ω-5 gliadin	LLAMAMNIASASRLLSPRGKEL HTPQEQFPQQQ	9-41	3707.91
ω-5 gliadin	ASRLLSPRGKELHTPQEQFPQQ QQFPQPQQFPQ	19-51	3901.98
ω-5 gliadin	QQFPQQEFPQQQQFPQQQIARQ PQQLPQQQQIP	221-253	4043.02
ω-5 gliadin	PQQQQIPQQPQQFPQQQFPQQQ QFPQQQEFPQQ	299-331	4090.91
ω-5 gliadin	GKELHTPQEQFPQQQQFPQPQQ FPQQQIPQQH	27-58	3879.90
ω-5 gliadin	SRLLSPRGKELHTPQEQFPQQQ QFPQPQQFP	20-50	3704.87
	QQFPQQQFPQQKLPQQEFPQQQ ISQQPQQLP		

ω-5 gliadin	QFPRPQQSPEQQQFPQQQFPQQ PPQQFPQQQ	112-142	3777.85
ω-5 gliadin	QQQIPQQPQQFLQQQQFPQQQP PQQHQFPQQ	374-404	3806.78
ω-5 gliadin	FPQQQPPQQHQFPQQQLPQQQQ IPQQQQIP	144-174	3839.83
ω-5 gliadin	QIPQQQQIPQQPQQFPQQQFPQQ QFPQQQF	160-189	3645.82
ω-5 gliadin	IPQQQQIPQQPQQIPQQQQIPQQ PQQFPQ	194-223	3723.76
ω-5 gliadin	QQIPQQPQQFPQQQFPQQQFPQ QQFPQQE	182-210	3477.72
	FIIFVLLAMAMNIASASRLLSPR GKELH		
ω-5 gliadin	QQQIPQQQIPQQHQIPQQPQQFP QQQQF	199-227	3608.72
ω-5 gliadin	QQPQQFPQQQFPQQQFPQQQFP QQEFPQ	4-31	3114.76
ω-5 gliadin	LPQQQQIPQQQIPQQPQQIPQQ QQIP	76-103	3451.71

ω-5 gliadin	QQFPQQQFPQQQLPQKQFPQP QQIP	203-230	3516.62
ω-5 gliadin	PQQPPQQFPQQQFPIPYPPQQSE EP	176-202	3215.72
ω-5 gliadin	FPQQQFPQQKLPQQEFPQQQISQ QP	274-299	3190.60
ω-5 gliadin	PQQQQFPQQQFPQQQQLPQKQF PQP	393-417	2961.41
ω-5 gliadin	QQFPQQQQFPQQHQSPQQQFPQ QQ	114-138	3054.52
	QQSPQQQQFPQQQFPQQQQLP		
ω-5 gliadin	PQQPQQFPQQQQFPQQQSPQQ	271-295	3076.53
ω-5 gliadin	QPQQFPQQQIPQQHQIPQQPQ	95-118	2994.41
	PQQPQQFPQQQQFLQQQIPQ		
ω-5 gliadin	PQQQIARQPQQLPQQQQIPQ	268-288	2568.22
ω-5 gliadin	QQQFPIPYPPQQSEEPSPYQ	253-273	2550.21
ω-5 gliadin	IPQQPQQFPQQQQFPQQQSP	45-65	2552.25

ω-5 gliadin	QIPQQQQIPQQPQQIPQQQQIPQ QPQQFPQQQFPQQQFP	61-81	2594.26
ω-5 gliadin	PQQFPQQQIPQQHQIPQQP	235-254	2384.24
ω-5 gliadin	QLPQKQFPQPQQIPQQQQI	402-421	2389.11
ω-5 gliadin	QFPQQEFPQQQQFPQQQIA	252-271	2407.20
ω-5 gliadin	QLPQQQFPQQQFPQQQFPQ	181-219	4732.39
ω-5 gliadin	QQPQQFPQQQFPQQQQFPQ	46-64	2296.14
ω-5 gliadin	QQQLPQQQFPQQQFPQQQF	286-304	2302.21
ω-5 gliadin	PQPQQFPQQQIPQQHQIP	222-240	2347.10
ω-5 gliadin	QQQIPQQQIPQQHQIPQ	342-360	2371.16
ω-5 gliadin	QQFPQQQQLTQQQFPRPQQSPE QQQFPQQQFPQ	306-324	2385.15
ω-5 gliadin	QLPQQQQIPQQQQIPQQPQQIPQ QQQIPQQP	340-358	2401.14
ω-5 gliadin		44-61	2167.10
ω-5 gliadin	QQQQFPQQQFPQQQQLPQKQFP QPQQIPQQQ	76-92	2069.03

ω-5 gliadin	LPQQEFPQQQISQQPQQLPQQQ QIPQQPQQ	62-394	4081.94
ω-5 gliadin	PQQQQIPQQPQQFLQQQQFPQQ QPPQQHQF	175-205	3697.89
	PQQFPQQQIPQQHQI		
ω-5 gliadin		272-302	3830.87
	QPQQFPQQQFPQQQF		
ω-5 gliadin		124-153	3593.8
	KLPQQEFPQQQISQQPQQLPQQ QQIPQQP		
ω-5 gliadin	PQQFPQQQIPQQHQIPQQPQQFP QQQQFL	142-171	3712.80
ω-5 gliadin	QQPQQIPQQQQIPQQPQQFPQQ QFPQQQF	46-60	1845.91
ω-5 gliadin		204-218	1903.90
	PQQQQIPQQPQQIPQQQQIPQQP QQFPQ		
ω-5 gliadin		123-151	3465.79
	QFPQQQQFPQQQSP		
ω-5 gliadin	HQFPQQQLPQQQQI	46-74	3570.71
	PQQFPQQQQFPQQH		
ω-5 gliadin		190-218	3574.75
	LLSPRGKELHTPQEQFPQQQQFP QPQQ		

ω-5 gliadin	IPQQPQQIPQQQQIPQQPQQFPQ QQFP	183-210	3364.64
ω-5 gliadin	FPQQQQFPQQQQLTQQQFPRPQ QSP	258-271	1715.80
ω-5 gliadin	PQQSEEPSPYQQ	169-182	1750.84
ω-5 gliadin	QQFPQQQIARQP	94-107	1766.81
ω-5 gliadin	QQQQLTQQQFPR	22-48	3217.57
ω-5 gliadin	IPQQQQIPQQP QFLQQQQIPQQ	188-214	3253.64
ω-5 gliadin	QFPQPQQIPQQQQIPQQPQQFPQ QQFPQQQQ	358-382	3069.49
ω-5 gliadin	PQQEFPQQQI	411-422	1419.60
ω-5 gliadin	PQQQISQQPQQLPQQQQIP	232-243	1468.75
ω-5 gliadin	PQQPQQFPQQQQFPQQQSPQQQ QFPQQ	366-377	1531.75
ω-5 gliadin	TPQEQFPQQQQFPQPQQFPQQQI PQQHQIPQQPQ	298-308	1287.68

ω-5 gliadin		72-82	1388.67
	LTQQQFPRP		
ω-5 gliadin		291-321	3799.83
	QQQFHQQQL		
ω-5 gliadin	QQIPQQPQQFPQQQFPQQQFPQ QQFPQQEFPQ	125-134	1242.61
ω-5 gliadin		130-148	2242.17
	QQQIPQQQIPQQHQIP		
ω-5 gliadin		253-279	3306.55
	NIASASRL		
ω-5 gliadin	KTFIIFVLLAMAMNI	32-65	4136.04
	PQQQQFPQQHQSPQQ		
ω-5 gliadin		370-378	1114.60
	QLPQQQQIPQQQQIPQQPQQIP		
ω-5 gliadin		335-343	1185.57
	QPPQQFPQQQFPIPYPPQQSEE		
ω-5 gliadin		199-230	3982.87
	QPQQFPQQQIPQQHQIPQQPQQ		
ω-5 gliadin	IPQQPQQIPQQQQIPQQPQQFPQ QQFPQQ	76-91	1940.98
ω-5 gliadin		15-22	831.48
	PQQQQFPQQQFPQQQQLPQKQF PQPQQIPQ		
ω-5 gliadin		2-16	1725.98

	PQQIPQQQQIPQQPQQFPQQQFP		
ω-5 gliadin		98-112	1834.86
	PQQFPQQQFPIPYPPQ		
ω-5 gliadin		175-196	2621.38
	QPQQLPQQQQ		
ω-5 gliadin		395-416	2638.27
	LLSPR		
ω-5 gliadin		45-66	2680.31
	QQIA		
ω-5 gliadin		188-216	3510.72
	ELHT		
ω-5 gliadin	PQQK	271-300	3670.84
	QQQIPQQQIP		
ω-5 gliadin		192-214	2787.38
	QQQIA		
ω-5 gliadin		397-412	1940.95
	QIPQ		
ω-5 gliadin		137-146	1225.58
	PQ		
ω-5 gliadin		22-26	585.37
ω-5 gliadin		237-240	460.24
ω-5 gliadin		29-32	499.25

ω-5 gliadin	120-123	502.25
ω-5 gliadin	76-85	1209.62
ω-5 gliadin	236-240	587.32
ω-5 gliadin	53-56	485.27
ω-5 gliadin	33-34	244.13

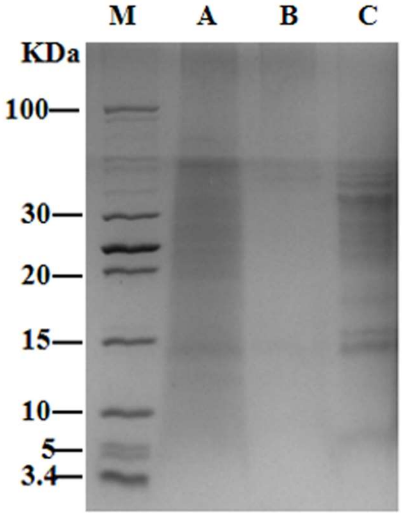

Figure S1. Tricine-SDS-PAGE of gastrointestinal digestion products of gliadin. M:Marker; A: gastric digestion products; B:intestinal digestion products; C: gliadin.

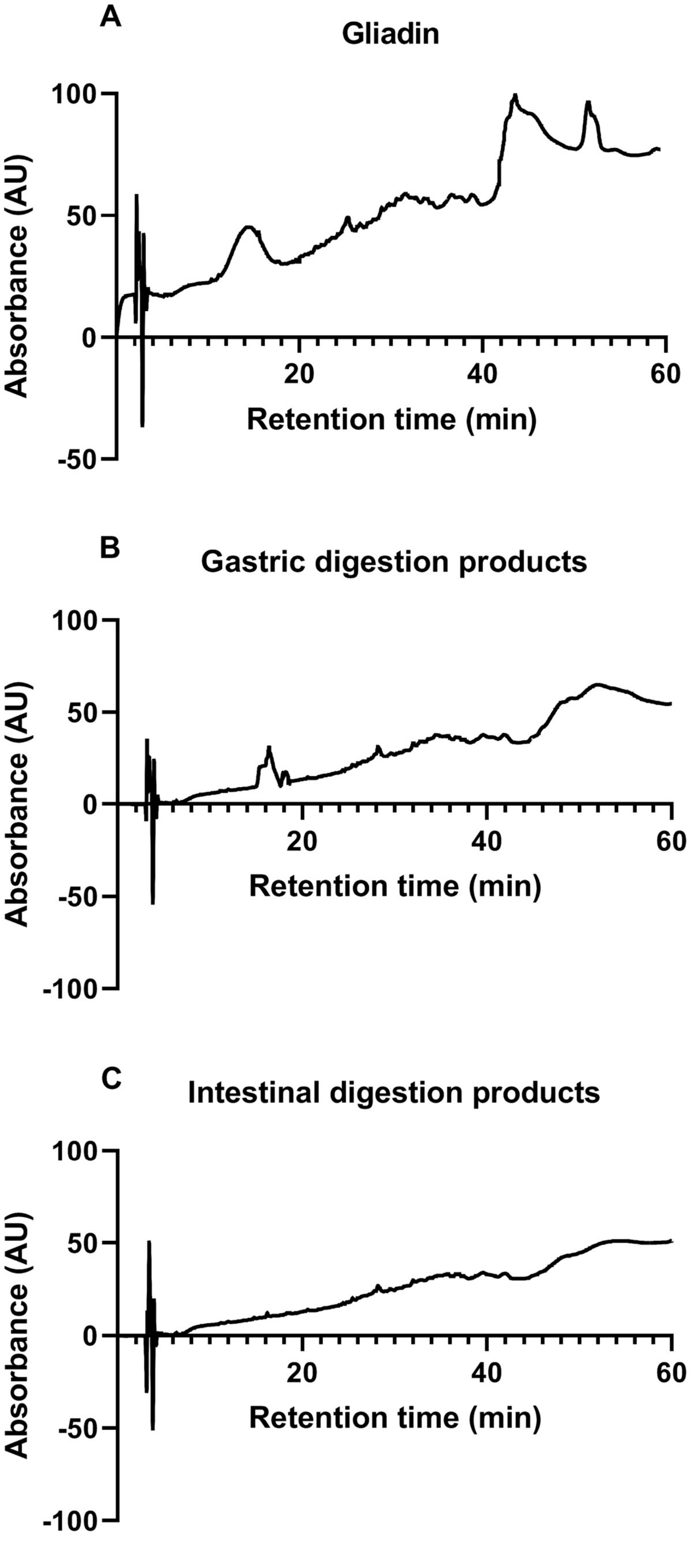

Figure S2. RP-HPLC chromatogram of gastrointestinal digestion products of gliadin.

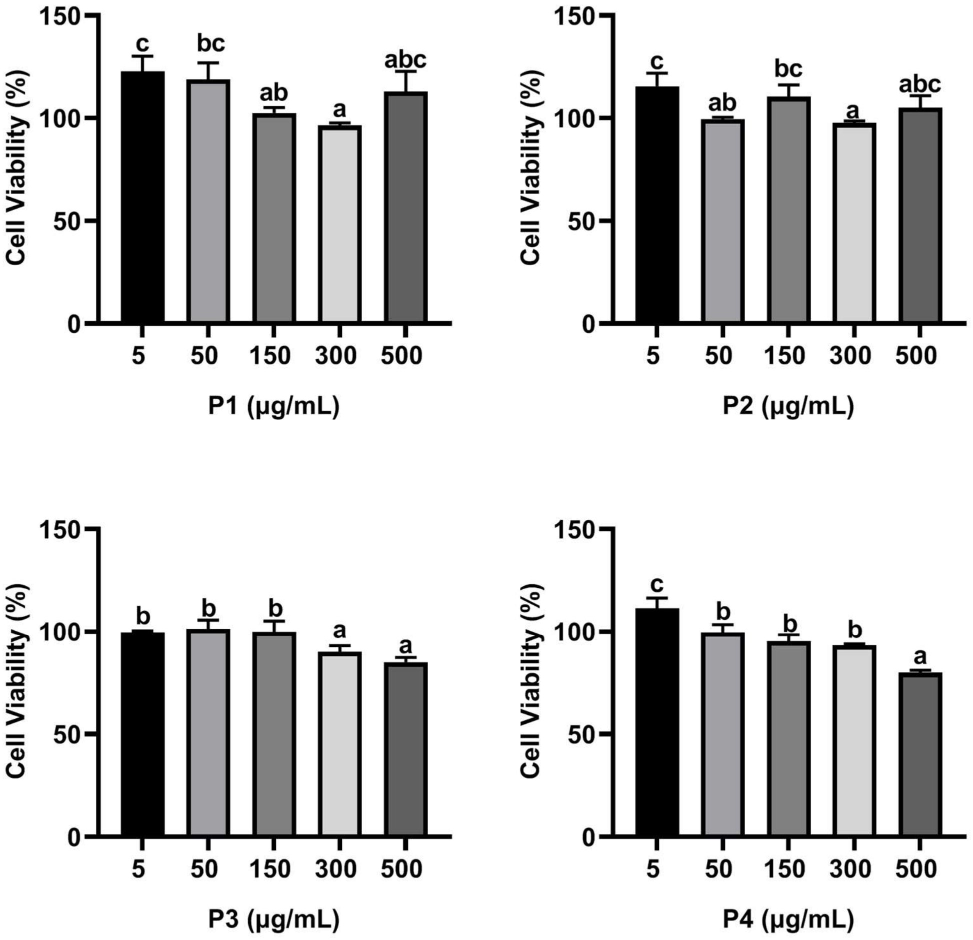

Figure S3. Effect of ω-5 gliadin -derived peptides on the viability of KU812 cells.

Note: Different letters indicate significant differences between groups (P<0.05).

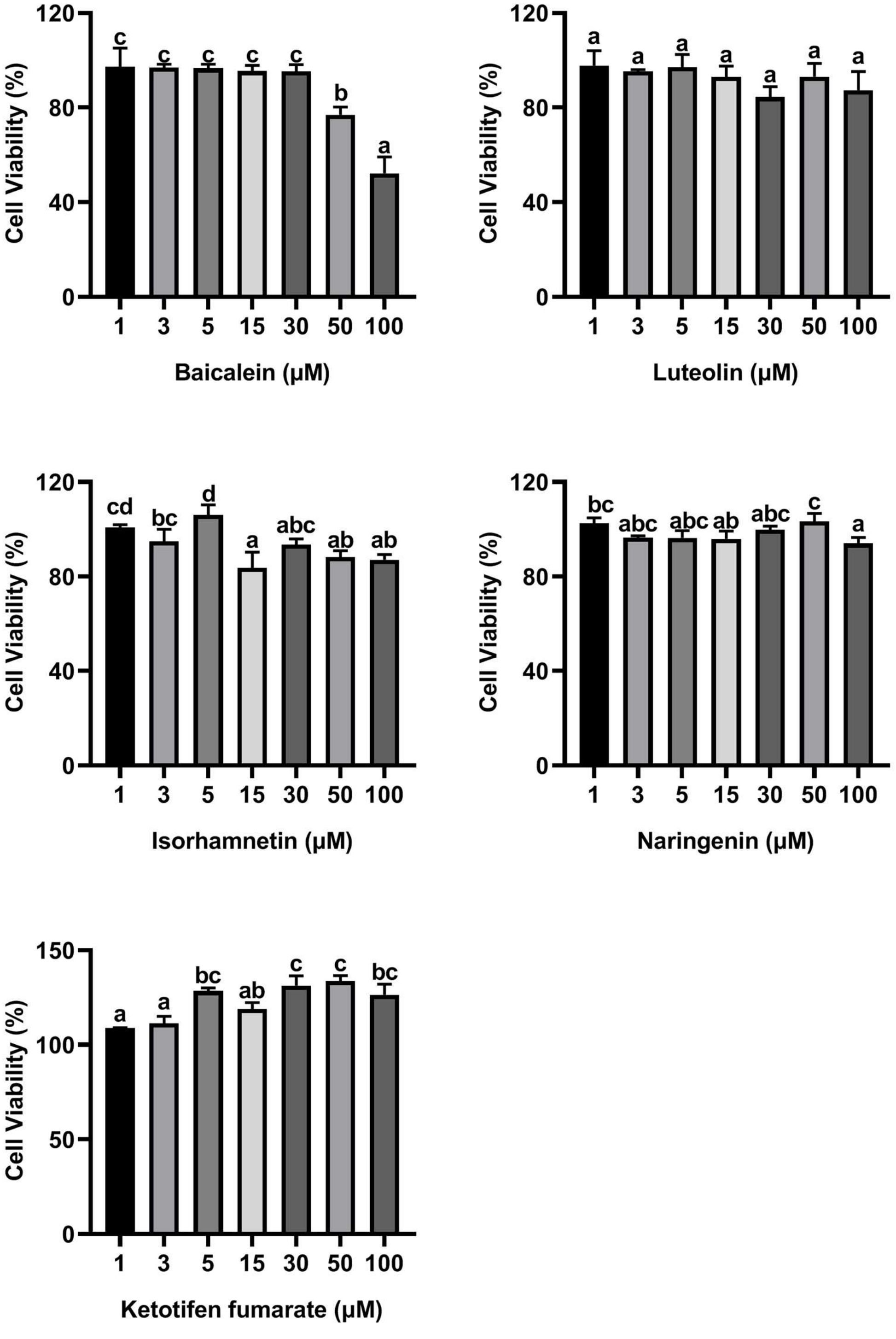

Figure S4. Effect of four flavonoids and ketotifen fumarate on the viability of KU812 cells.

Note: Different letters indicate significant differences between groups ($P<0.05$).

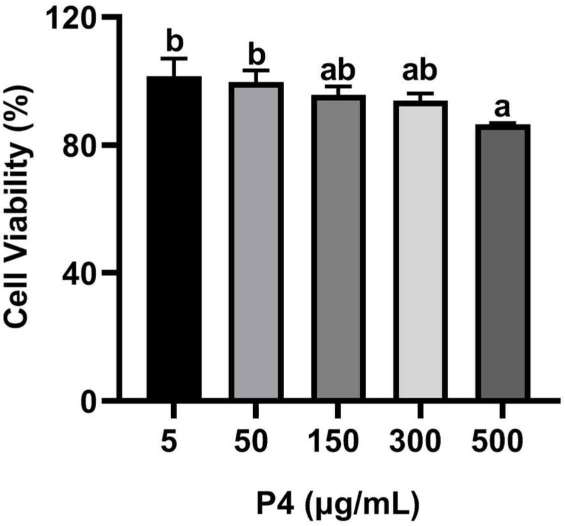

Figure S5. Effect of ω-5 gliadin-derived peptides P4 on the viability of Caco-2 cells.

Note: Different letters indicate significant differences between groups (P<0.05).

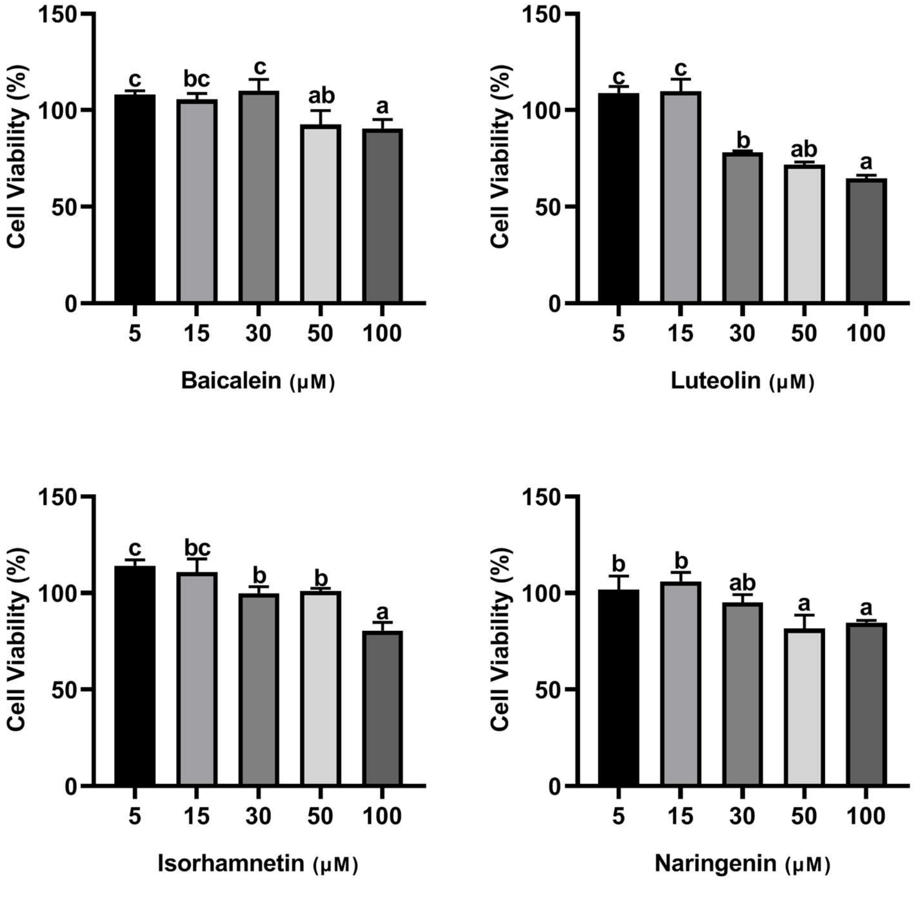

Figure S6. Effect of four flavonoids on the viability of Caco-2 cells.

Note: Different letters indicate significant differences between groups (P<0.05).

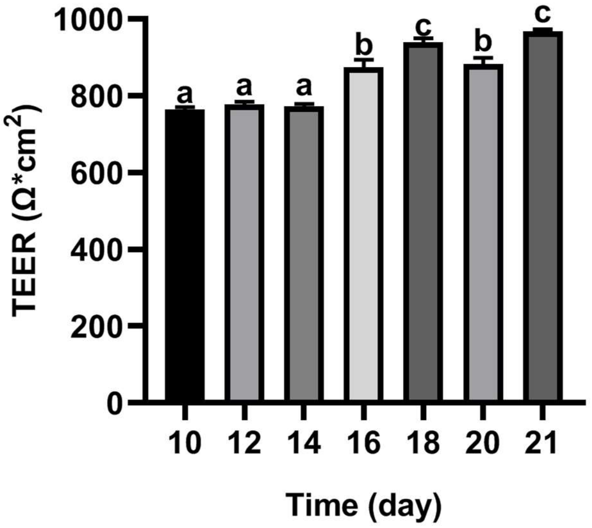

Figure S7. Determination of TEER of the Caco-2 cell monolayer.

Note: Different letters indicate significant differences between groups (P<0.05).
